# An Agent-Oriented Framework for CityGML Querying: Retrieval and Deterministic Workflow Evaluation

**DOI:** 10.3390/s26216817

**Published:** 2026-10-28

**Authors:** Phuoc-Dat Lam, Ngoc-Giao Pham, Jiyoung Oh, Soo-Yol Ok, Suk-Hwan Lee

**Affiliations:** 1 Department of Computer Engineering, Dong-A University, Busan 49315, Republic of Korea; datlam10006@gmail.com; 2 Department of Computer Fundamentals, FPT University, Hanoi 10000, Vietnam; giaopn4@fe.edu.vn; 3 College of Interdisciplinary Studies, Dong-A University, Busan 49315, Republic of Korea; ohjy0105@dau.ac.kr; 4 Center for Food & Bio Innovation, DAU G-LAMP Project Group, Dong-A University, Busan 49315, Republic of Korea

**Keywords:** digital twin, CityGML, metadata retrieval, agent-oriented framework, agent coordination, structured metadata, 3D building models

## Abstract

Querying CityGML building models requires access to structured semantic information and a clear distinction between retrieved evidence and answer correctness. This study presents an agent-oriented framework combining metadata extraction, deterministic query coordination, evidence validation, and bounded recovery. Evaluation uses four static CityGML 2.0 building models containing LoD4 geometry and 4,181 processed metadata entities. A standalone retrieval experiment compares BM25 and dense E5 using 80 queries, while a separate 32-query benchmark compares direct metadata processing, a workflow without validation and recovery, and the full workflow against independently reconstructed reference answers. BM25 achieved overall Precision@5 of 0.5225, Recall@5 of 0.5325, and MRR@10 of 0.9150, outperforming dense E5 under the evaluated representation. Median retrieval latency was 4.80 ms for BM25 and 68.10 ms for dense E5. Analysis by query type and reference-set size distinguished ranking performance from the attainable Recall@5 ceiling; document truncation also removed query-defining information from some inputs. Direct processing and the ablated workflow each produced 32 correct final outcomes, compared with 26 for the full workflow, whose validation and recovery increased latency without improving correctness on this workload. Metadata preprocessing required approximately 1.47–15.15 s. These results inform the design of CityGML query workflows by distinguishing retrieval coverage, structured-result correctness, and final-answer reliability.

## 1. Introduction

The rapid urbanization of modern cities has significantly increased the complexity of urban systems [1], including buildings, transportation networks [2], utilities, and  disaster management infrastructures [3]. To effectively manage this complexity, Digital Twin (DT) technology has emerged as a key paradigm for smart cities, enabling the virtual representation of physical urban environments and supporting data-driven analysis, simulation, and  decision-making [4,5]. In this context, 3D city models play a crucial role as the geometric and semantic foundation of urban digital twins.

Among existing standards, City Geography Markup Language (CityGML) has been widely adopted for representing 3D city models with rich semantic hierarchies and multiple Levels of Detail (LoD) [6]. CityGML enables standardized modeling of buildings, terrain, transportation, and city furniture, making it suitable for interoperability across urban applications such as disaster response, urban planning, and infrastructure management [7,8,9]. However, the complexity and large scale of CityGML datasets pose challenges for efficient data access, semantic understanding, and user interaction, especially for non-expert users [10,11].

Recent advances in Large Language Models (LLMs) offer new opportunities for intelligent interaction with complex spatial data [12,13]. In particular, Retrieval-Augmented Generation (RAG) enhances LLMs by integrating external knowledge sources, enabling accurate, context-aware, and explainable responses grounded in domain-specific data [14]. Applying RAG to CityGML-based digital twins allows users to query, analyze, and reason about 3D city models using natural language, bridging the gap between complex urban data and human-centered decision-making [15,16,17].

This study presents an agent-oriented framework for querying static CityGML building models. The implemented prototype combines structured metadata extraction, deterministic query routing, evidence checks, and response construction. A separate retrieval experiment compares BM25 and Dense E5 across four CityGML 2.0 building models containing LoD4 geometry. The framework is intended as a potential query component for future digital-twin applications; the present implementation does not ingest sensor observations, synchronize physical and digital states, or evaluate time-dependent conditions.

The main contributions are:

- An entity-level metadata representation for querying CityGML building models.
- A deterministic coordination workflow with explicit evidence checks and bounded follow-up actions.
- A four-building evaluation of metadata retrieval, query latency, coordination behavior, and preprocessing resource requirements.

## 2. Related Work

### 2.1. City Geography Markup Language

City Geography Markup Language (CityGML), standardized by the Open Geospatial Consortium (OGC) [6], is one of the most widely used data models for representing and exchanging 3D city models. CityGML provides a semantically rich representation of urban objects, including buildings, bridges, roads, land use, and  vegetation, organized into thematic modules and hierarchical object classes [6,18]. CityGML 2.0 supports five Levels of Detail (LoD0–LoD4), ranging from coarse representations to detailed indoor modeling, enabling applications ranging from large-scale urban visualization to detailed indoor modeling [19,20,21].

Previous studies have applied CityGML to urban visualization, building information integration, and domain-specific 3D city modeling [22,23]. Extensions such as Application Domain Extensions (ADEs) have been proposed to enrich CityGML with domain-specific attributes [24,25,26]. Despite these advantages, CityGML datasets are often represented using complex XML-based structures, making them difficult to query and analyze efficiently. Consequently, many studies have focused on transforming CityGML data into databases, graph models, or semantic knowledge representations to improve usability and performance [27,28].

CityGML 3.0 extends the modeling context beyond the CityGML 2.0 datasets used in this study. Its conceptual model supports indoor and outdoor representations within LoD0–LoD3, rather than assigning indoor modeling exclusively to LoD4 [29]. IndoorGML addresses a complementary concern by representing indoor spaces and their topological relationships for navigation [30]. These distinctions are relevant when separating building semantics and geometry from navigable connectivity. The present evaluation uses CityGML 2.0 metadata and does not establish compatibility with CityGML 3.0 or validated IndoorGML navigation models.

Database-based infrastructure provides another approach to managing semantic city models. 3DCityDB supplies a spatial database schema and tools for importing, exporting, managing, and analyzing CityGML data  [31]. Such infrastructure differs from the canonical metadata artifacts and in-memory retrieval structures evaluated here. 3DCityDB is therefore relevant as an established data-management approach, although it was not included as an experimental baseline in this study.

### 2.2. Retrieval-Augmented Generation

Retrieval-Augmented Generation (RAG) is an emerging approach that combines information retrieval techniques with generative language models [32,33,34]. Instead of relying solely on the internal knowledge of an LLM, RAG retrieves relevant documents, structured data, or embeddings from external knowledge sources and uses them as contextual information for response generation. This approach improves factual accuracy, reduces hallucination, and enables domain-specific reasoning [35,36].

RAG has been widely applied in domains such as question answering, technical document analysis, healthcare, and scientific research [37,38,39,40]. Typical RAG pipelines involve document preprocessing, embedding generation, vector database indexing, similarity-based retrieval, and LLM-based answer synthesis [41,42]. Recent studies have also explored agent-based RAG architectures, in which multiple specialized agents collaborate to perform tasks such as retrieval, reasoning, verification, and explanation [35,37,41,43].

However, most existing RAG applications focus on unstructured textual data. The application of RAG to spatial and semantic 3D data, such as CityGML, remains relatively underexplored. Key challenges include representing geometric information in a retrievable form, preserving semantic relationships, and aligning spatial data with natural-language queries.

### 2.3. Natural-Language Interfaces to CityGML and Geospatial Data

Several recent studies have attempted to integrate LLMs with geospatial data for intelligent urban analysis. Existing approaches include natural-language interfaces for GIS databases, knowledge graph-based urban reasoning, and semantic querying of 3D city models. Some studies transform CityGML data into relational databases, RDF graphs, or knowledge graphs to support semantic querying and reasoning [44,45].

Building upon these efforts, a CityGML RAG system represents a novel integration of CityGML-based digital twins with retrieval-augmented language models [46]. In such systems, CityGML objects and their attributes are parsed, semantically structured, and indexed using vector representations. When a user submits a natural-language query, the system retrieves relevant city objects or spatial contexts and generates responses grounded in the underlying 3D city model [47].

Compared with traditional query-based systems, CityGML RAG systems offer greater flexibility, interpretability, and accessibility. The  use of a multi-agent architecture further enhances scalability and task decomposition, enabling individual agents to independently handle geometric analysis [48], semantic retrieval [49], disaster reasoning [50], and explanation generation [51]. Nevertheless, research in this area remains at an early stage, and comprehensive frameworks tailored to smart city digital twins remain limited.

Recent studies have explored LLM-based interfaces for accessing geospatial data through natural-language queries. These interfaces translate user requests into executable code, select geospatial processing tools, and coordinate spatial analysis workflows  [52,53]. In urban digital-twin applications, LLM interfaces have also combined CityGML building information with sensor observations to support geospatial and temporal queries [46]. These approaches extend language-based interaction beyond document retrieval to structured queries and tool execution. The present study focuses on metadata retrieval and deterministic query coordination over static CityGML building models, while live LLM summarization remains optional and unevaluated.

This study investigates an agent-oriented query workflow for static CityGML building models and evaluates its metadata-processing components. Its relevance to urban digital twins concerns potential reuse as a query layer, rather than demonstrated sensor integration or operational disaster-response capability.

## 3. Proposed Framework

**Figure 1:**
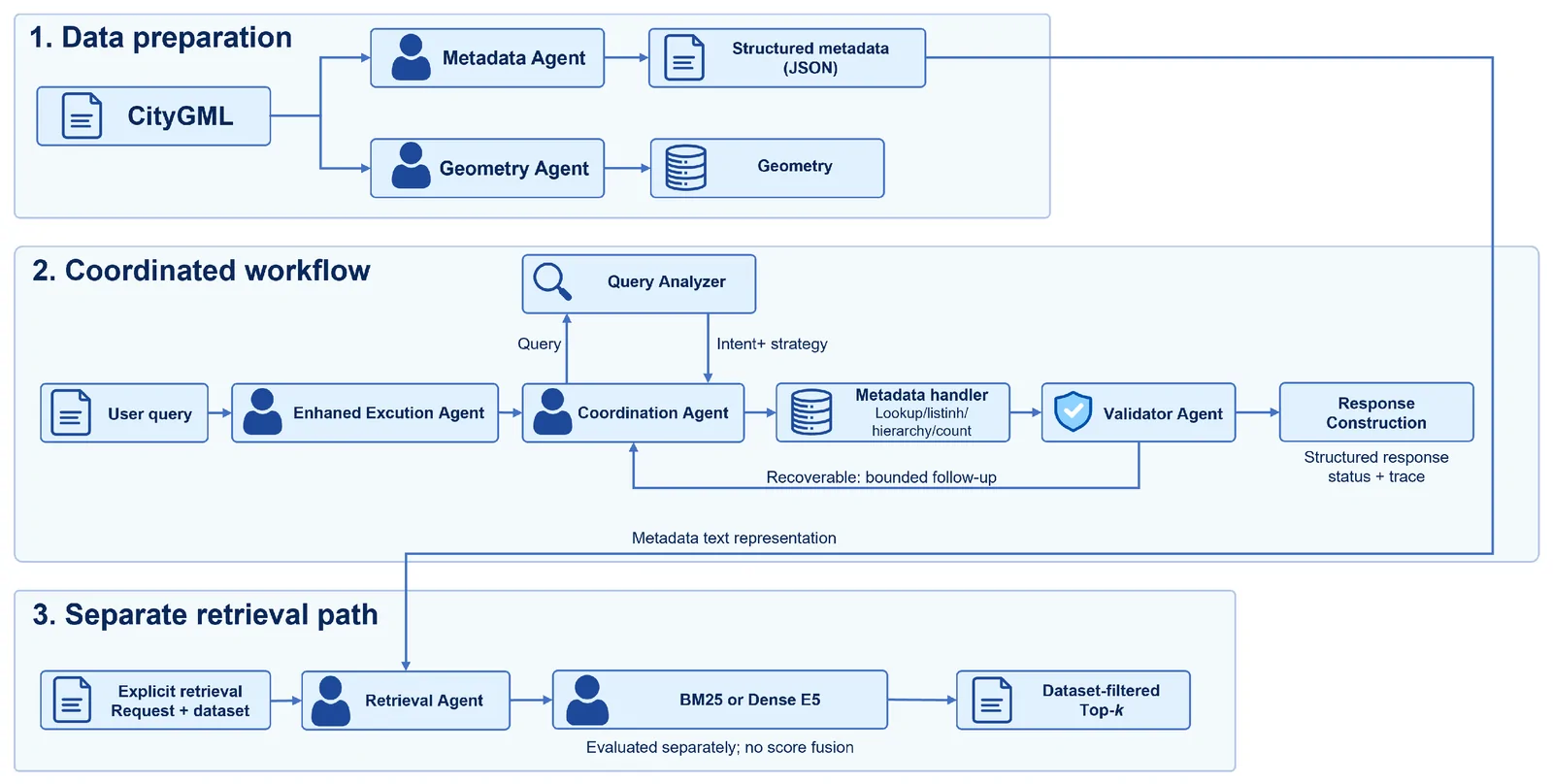
CityGML query framework comprising data preparation, a coordinated metadata workflow, and a separate retrieval path. Structured metadata supports both direct metadata processing and BM25 or Dense E5 retrieval, evaluated separately without score fusion. The coordinated workflow includes intent analysis, evidence validation, bounded follow-up, and response construction. Geometric operations await independent validation; optional LLM summarization is omitted and was not evaluated.

This section presents an agent-oriented framework for querying static CityGML building models. As illustrated in Figure 1, the framework comprises three parts: (1) CityGML parsing and data preparation, (2) coordinated metadata query processing, and (3) a separate metadata retrieval path. The preparation stage extracts structured metadata and geometric information. The coordinated workflow performs intent analysis, strategy-specific metadata execution, evidence validation, bounded follow-up, and deterministic response construction. The retrieval path evaluates BM25 and Dense E5 separately using dataset-filtered ranking. Geometric operations and the optional language-model interface are described with their current validation scope in Section 3.4.5 and Section 3.4.6, respectively.

### 3.1. Problem Definition and System Overview

Let a static CityGML model be represented as a structured collection of *n* objects,

$$C=\{O_{1},O_{2},\ldots ,O_{n}\},$$

where C denotes the collection, *n* is the number of objects, and each object $O_{i}$, for i=1,…,n, represents a semantic entity such as a building, room, window, or door. The collection describes the available model snapshot; it does not include a time-varying state or a synchronization mechanism with the physical building.

Given a natural-language query *Q*, the objective of the system is to retrieve a relevant subset of city objects and generate a grounded response

F:Q→R,

where *R* denotes a semantically grounded and explainable response constructed from the selected static CityGML model.

### 3.2. 3D CityGML Parsing and Data Preparation

#### 3.2.1. CityGML Object Representation

Each CityGML object $O_{i}$ is represented as a tuple:

(1) $$O_{i}=\left(g_{i},s_{i},m_{i}\right)$$

where:

- $g_{i}$ denotes the available geometric information, such as vertices, polygonal surfaces, and bounding volumes;
- $s_{i}$ denotes the semantic information (e.g., type, attributes, relationships)
- $m_{i}$ denotes supplementary attributes available in the source data; material descriptions do not by themselves establish a component’s fire-resistance rating.

#### 3.2.2. Metadata Parsing via Metadata Agent

The Metadata Agent extracts entity-level information from CityGML XML files using deterministic parsing rules. For the eight supported entity types listed in Section 4.1.1, it records the entity identifier, type, available semantic fields, generic attributes, and parent building or building-part identifiers. The parsing operation is expressed as:

(2) $$P:\mathrm{XML}\to M_{D},$$

where $M_{D}$ denotes the canonical metadata collection for dataset *D*.

The extracted records are normalized and stored in a dataset-specific JSON artifact. Available parent identifiers preserve containment context, while material descriptions are retained as metadata without inferring fire-resistance properties. The resulting collection supports direct entity lookup, listing, counting, and attribute queries in the coordinated workflow. It also supplies the entity-level text representation described in Section 3.3.1. Geometry extraction is performed separately by the Geo Agent.

#### 3.2.3. Geometric Parsing via Geo Agent

The Geo Agent extracts geometric information from CityGML objects for candidate spatial operations. Geometry is represented separately from the canonical metadata produced by the Metadata Agent. For an object $O_{i}$ with available geometry, its geometric component is expressed as:

(3) $$g_{i}=\left\{V_{i},F_{i},B_{i}\right\}$$

where:

- $V_{i}=\left\{v_{1},v_{2},\ldots ,v_{m}\right\}\subset R^{3}$ represents the set of vertices defining the 3D geometry;
- $F_{i}$ denotes the set of polygonal faces formed by connecting vertices in $V_{i}$;
- $B_{i}$ denotes the spatial boundary or bounding volume of the object.

These representations provide inputs for candidate adjacency and connectivity analysis and graph-based routing. Extracted geometry alone does not establish traversable connections or physically feasible routes. The implemented operations, coordinate assumptions, geometric tolerances, and current validation limitations are described in Section 3.4.6. Geometry extraction and spatial operations were excluded from the reported metadata preprocessing and query experiments.

### 3.3. Metadata Representation and Retrieval

#### 3.3.1. Entity-Level Text Representation

For the measured retrieval experiment, each metadata entity is represented by one text document containing its dataset identifier, entity type, GML identifier, and available semantic fields: name, description, class, function, and usage. Parent building and building-part identifiers are included when available, followed by generic attributes sorted by key. Duplicate generic-attribute details are excluded.

Each entity remains a single document, without overlapping text windows. Documents are truncated at 4096 characters. The resulting corpus contains 4181 documents, of which 1133 were truncated. Hierarchical context is represented only by the available parent identifiers; ancestor paths, child expansion, and graph representations are not included. Truncation may remove attributes occurring near the end of a document and should be considered when interpreting retrieval effectiveness.

#### 3.3.2. Dataset-Filtered BM25 Retrieval

The measured retrieval backend uses BM25 with $k_{1}=1.5$ and b=0.75. Text is lowercased and tokenized using Unicode word matching. Document statistics are computed over the shared corpus, whereas retrieval candidates are restricted to the specified dataset. The backend returns the ten highest-ranked candidates, from which the retrieval metrics in Section 4 are computed.

BM25 and the dense retrieval condition described in Section 3.3.3 use the same locked corpus, benchmark queries, and reference relevance sets. This shared evaluation setup supports comparison of lexical and embedding-based retrieval under dataset filtering. The two methods are evaluated separately; no score fusion or hybrid ranking is applied. Retrieval effectiveness and latency are reported in Section 4.3 and Section 4.4.1, respectively.

#### 3.3.3. Dense Embedding Retrieval

A dense retrieval condition was implemented using intfloat/multilingual-e5-base, with model revision 835193815a3936a24a0ee7dc9e3d48c1fbb19c55. The model produces 768-dimensional embeddings and was executed locally on the CPU. Queries and metadata documents were prefixed with “query:” and “passage:”, respectively. Inputs were limited to 512 tokens using right-side truncation, and the resulting embeddings were L2-normalized. Retrieval used exact cosine similarity, implemented as the dot product between normalized float32 vectors. Candidates were restricted to the selected dataset before selecting the ten highest-ranked documents.

BM25 and dense retrieval used the same locked corpus and query-specific reference relevance sets. The document representation was capped at 4096 characters before embedding; the dense encoder additionally applied its token limit. No retrieval parameters were selected using the reported test results. The previously configured Nomic embedding model was not evaluated.

### 3.4. Agent Coordination and Query Execution

The implemented workflow separates query analysis, execution control, evidence validation, and response construction. These components operate through deterministic rules in the reported metadata experiments. The entry point is EnhancedExecutionAgent, which passes the query and request context to CoordinationAgent. The coordinator selects a processing strategy, executes the corresponding handler, and submits its output to ValidatorAgent. The standalone ExecutionAgent class is a legacy alternative and was not used in these measurements. Algorithm 1 summarizes the deterministic metadata query workflow, including intent analysis, plan selection, metadata execution, evidence validation, bounded recovery, and response construction with execution tracing.

The separate BM25 retrieval experiment follows a different execution path: an explicit metadata-retrieval context dispatches directly to RetrieverAgent, bypassing query analysis, coordination, and workflow validation. Accordingly, its retrieval metrics and the coordination measurements characterize distinct execution paths.
**Algorithm 1** Deterministic metadata query workflow with execution tracing**Input**: Query *Q*, selected dataset *D*, output format *F*, configured recovery limit *K* **Output**: Structured response *R* and execution trace *T*
  1:Load canonical metadata $M_{D}$ for dataset *D*  2:Initialize metadata workflow components  3:Initialize query context $S\leftarrow \{D,M_{D}\}$  4:Initialize execution trace T←∅  5:Initialize recovery count k←0  6:Initialize evidence E←∅  7:Initialize status σ←PENDING  8:I←ANALYZEINTENT(Q)  9:P←SELECTMETADATAPLN(I,S)10:**if** no supported metadata plan is available **then**11:    σ←UNSUPPORTED12:**else**13:      **while** σ=PENDING **do**14:            (E,e)←EXECUTEMETADATAPLAN(P,Q,S)15:            Record instrumented component entries and calls in *T*16:            **if** *e* indicates an execution error **then**17:                  σ←EXECUTION_FAILURE18:                  Record error details in *T*19:            **else**20:                  V←VALIDATEWORKFLOWEVIDENCE(Q,E,S)21:                  Record validator entries and outcome in *T*22:                  **if** *V* accepts the returned evidence **then**23:                         σ←COMPLETED24:                  **else if** *V* permits recovery and k<K **then**25:                        $P^{\prime }\leftarrow SELECTRECOVERYPLAN\left(P,V,S\right)$26:                        **if** $P^{\prime }$ preserves the operation and its arguments **then**27:                             Record a same-operation retry in *T*28:                        **else**29:                              Record a substantive plan revision in *T*30:                        **end if**31:                        $P\leftarrow P^{\prime }$32:                        k←k+133:                  **else**34:                        σ←INSUFFICIENT_EVIDENCE35:                  **end if**36:            **end if**37:      **end while**38:**end if**39:R←FORMATDETERMINISTICRESPONSE(E,σ,F)40:Attach dataset identifier and final status to *T*41:**return** (R,T)


#### 3.4.1. Query Analysis and Strategy Selection

The Query Analyzer receives a query string and returns a structured intent containing the query type, entities, requested attributes, filters, spatial criteria, confidence, and processing strategy. Classification uses ordered regular-expression rules with the following first-match priority: door proximity, spatial, hierarchy, building-specific, vulnerability, metadata, statistical, and general queries.

The selected category determines a processing strategy. Building-specific queries use direct lookup; metadata queries use metadata analysis; hierarchy queries use hierarchy analysis; and statistical queries use aggregation. Spatial queries select a keyword-dependent proximity, neighborhood, navigation, or emergency strategy. Unmatched queries use general retrieval with a fixed confidence of 0.50. Analyzer exceptions use the same fallback with confidence 0.0. These confidence values are rule-assigned indicators, not calibrated probabilities.

Parameter extraction supports a limited set of numeric building and room identifiers, requested attributes, numeric thresholds, and spatial criteria. The detector does not select the declared building-search, material, or comparison categories through explicit branches. Furthermore, spatial rules match fire-related terms before vulnerability rules. These implementation limits restrict the supported query language; intent-classification accuracy was not independently evaluated.

#### 3.4.2. Coordination, Execution, and State

For each coordinated request, the coordinator creates an action plan from the selected strategy. Each action records its type, target, requested attributes or relations, constraints, dependencies, execution status, produced evidence, and attempt number. Request-local state contains the plan, accumulated evidence, execution trace, and follow-up counter. The selected dataset and loaded metadata are maintained by the dataset manager; changing the dataset refreshes the coordinator’s metadata snapshot and invalidates its geometry-agent instance.

The initial plan contains a primary action and a consistency-verification action. However, the initial executor invokes only one strategy-specific handler. It then collects the handler output and submits it to the validator. In the current implementation, all initial plan items are marked complete after this handler returns. Consequently, a completed consistency-verification plan item does not establish that a separate spatial consistency check was executed. Actual execution is determined from the event trace and executed follow-up actions.

Metadata handlers return structured results, source references, evidence, and deterministic answer text. The coordinator subsequently attaches validation results, plan information, execution status, processing metadata, and the execution trace. In experiment mode, exceptions from the coordinator are propagated rather than silently redirected to the legacy execution path.

#### 3.4.3. Evidence Validation and Bounded Recovery

The validator derives evidence requirements from the query and planned actions. Metadata actions require the requested attributes, whereas spatial and hierarchy actions require the corresponding relations. A consistency requirement is also associated with the verification action. The validator evaluates the active attribute, geometry, and consistency dimensions using configured weights of 0.34, 0.33, and 0.33, respectively, renormalized over the active dimensions.

A result is accepted only when its overall confidence is at least 0.80, all required evidence is available, and no critical unsupported assertion or conflict is detected. Explicitly unavailable entities, geometry, or topology can produce an unrecoverable deficiency. Otherwise, recommended follow-up actions or a confidence of at least 0.60 can produce a recoverable status. These thresholds are uncalibrated engineering defaults rather than empirically established correctness probabilities.

Follow-up actions depend on the detected deficiency. Missing attributes or empty retrieval can trigger semantic retrieval; missing spatial or hierarchical evidence can trigger the corresponding action; and unsupported assertions or conflicts can trigger consistency verification. The coordinator validates the initial result at attempt zero and permits at most two follow-up batches under the default configuration.

Action signatures include the attempt number, allowing the same operation to be retried in a later batch. A batch is classified as a same-operation retry when all scheduled actions have previously used base signatures; otherwise, it is classified as a revised plan. The internal revision counter increases for either case and therefore does not directly measure substantive strategy changes.

Execution terminates upon acceptance, an unrecoverable deficiency, exhaustion of the follow-up limit, or the absence of schedulable actions. Unresolved requests return an explicit insufficient-evidence response with verified=false. In metadata-only mode, semantic follow-up actions return no additional evidence. An unavailable geometry capability instead returns capability_unavailable, with validation marked as not run. Workflow acceptance denotes satisfaction of these internal checks, not independently measured answer correctness.

#### 3.4.4. Result Aggregation and Response Construction

The aggregation notation Φ denotes limited result assembly rather than a separately implemented agent or learned fusion operator. Evidence collection packages the non-empty handler output fields, excluding answer text and workflow bookkeeping, into an evidence record with available source references.

During follow-up execution, newly produced evidence is appended to the accumulated evidence, and semantic result dictionaries are appended to the result list. For geometry or route outputs, the most recent produced item replaces the previous geometric output. The response is then regenerated or formatted as appropriate.

This coordination-level aggregation does not deduplicate results, globally rank evidence, or reconcile conflicting values. Conflict detection belongs to validation and may request further verification; no conflict-resolution algorithm is implemented. Metadata lookup, listing, and count handlers construct their answers directly from structured metadata.

#### 3.4.5. Optional Language-Model Interface

The measured metadata workflow uses deterministic execution and does not invoke a language model. An optional live summarization path is available through SummarizerAgent and an Ollama-backed summarization tool, but this path was not exercised in the reported metadata measurements.

For reproducibility, the optional answer-generation path uses the following system message:

You are a CityGML evidence-grounded assistant. Use only the supplied evidence. Do not invent entity IDs, attribute values, geometry, topology, routes, or spatial relations. State when evidence is insufficient.

The corresponding user prompt is:

You are an expert in CityGML and urban data analysis.

Answer the following question based on the provided context.

Question: {query}

Context:

{context}

Provide a concise answer of no more than 80 words that:

1. Directly addresses the question

2. Is based on the provided context

3. Preserves relevant CityGML entity IDs and measured values

4. Acknowledges any limitations or assumptions

Answer:

When additional context is supplied, its string representation is inserted after the evidence context under the heading “Additional Context:”. The optional live call uses the configured temperature and output-token limit with think=False. These prompts document an optional interface and do not constitute evidence of live-model performance.

#### 3.4.6. Geometric Operations and Current Validation Status

The geometric extension distinguishes shared-boundary adjacency from door-mediated connectivity. Shared-boundary adjacency describes a geometric relationship between room boundaries, whereas door-mediated connectivity represents a candidate passage between rooms associated with the same door. A shared boundary alone is therefore insufficient to establish a traversable connection.

For the available D-Type geometry artifact, coordinates are interpreted in millimetres according to the source declaration XY-MM. Candidate generation uses a boundary tolerance of 50 mm, a minimum shared-boundary length of 100 mm, a door-to-room-boundary tolerance of 150 mm, and a floor-surface elevation tolerance of 20 mm. These thresholds are declared engineering assumptions; they have not been calibrated or validated against independently annotated spatial relationships. Their applicability to other datasets requires verification of coordinate systems and units.

Candidate connectivity is represented as a graph G=(V,E,w), where each node corresponds to a CityGML Room identifier. A bidirectional connection is proposed when the footprints of two rooms are both within the specified boundary-distance tolerance of the same door centroid. Each connection is represented by two directed edges with non-negative weights. The current edge weight is the two-dimensional Euclidean distance between room centroids, converted to metres. This weight is a geometric cost proxy and does not represent a validated indoor walking distance. Cross-floor edges require verified connector-to-room associations and are not inferred solely from proximity.

Dijkstra’s algorithm is implemented to find a minimum-cost path on the supplied graph, with an explicit no_path result when no connection exists. Optimality is defined with respect to the graph and its assigned weights; it does not establish the physical feasibility of a route. Routing to designated exits additionally requires independently confirmed exit labels. The system does not classify an arbitrary door as an emergency exit.

Candidate generation for D-Type produced 16 room nodes and 15 undirected connections, with no cross-floor connections or designated exits. These counts describe an unvalidated candidate graph rather than verified building topology. Independent room-pair labels, reference routes, and designated-exit annotations were unavailable. Consequently, adjacency accuracy, route feasibility, route-cost accuracy, and designated-exit routing success were not evaluated. Integration checks returned a blocked status for geometry requests requiring a validated graph. The geometric extension is therefore reported as an implemented capability awaiting independent validation, rather than as demonstrated geometric reasoning performance.

## 4. Results

### 4.1. Experimental Setup

The experimental evaluation uses four CityGML 2.0 building models containing LoD4 geometry and comprises three complementary measurements. First, a BM25 metadata retrieval baseline is evaluated using 80 unique queries, with 20 queries per model, to measure retrieval quality and online retrieval latency. Second, the deterministic metadata workflow is examined using a separate set of 32 unique queries, with eight queries per model, to characterize agent activations, instrumented calls, and retry behavior. Third, metadata preprocessing time, storage requirements, and process memory are measured for each model, together with the construction cost of the shared BM25 index.

These measurements characterize different components of the implementation. BM25 retrieval results describe the baseline retrieval stage, whereas coordination measurements describe the deterministic metadata workflow. Resource measurements cover metadata preparation and the explicitly identified query and indexing processes. The evaluation therefore provides component-level evidence across four building models; it does not constitute an end-to-end evaluation of live LLM generation, geometric reasoning, indoor navigation, or emergency-exit planning. The following subsections describe the datasets and workstation configuration, while the measurement boundaries are specified alongside the corresponding results.

#### 4.1.1. CityGML Models and Evaluation Scope

The dataset collection comprises four CityGML 2.0 building models containing LoD4 geometry: D-Type 106, A1–B1 107, C1 115, and SMARTCITY LAB. The models originate from IFC4x3 sources and contain semantic objects and detailed geometry for building-scale query processing. The SMARTCITY LAB source is the repository version containing 18 Room elements. This version is distinguished from alternative exports with different semantic inventories.

Table 1 summarizes the source-data characteristics. The models contain 16–21 rooms, 11–33 doors, and 16–33 windows, while their polygon counts range from 30,195 to 714,083. SMARTCITY LAB contains approximately 20.7 times as many polygon elements as D-Type 106 despite having a comparable room count. The collection therefore provides variation in geometric complexity as well as semantic object inventories. These characteristics describe individual building models and do not establish coverage of district- or city-scale datasets.

As summarized in Table 2, the canonical metadata contains 4181 entities across eight supported types: Building, BuildingPart, Room, WallSurface, Door, Window, BuildingInstallation, and FloorSurface. Dataset totals are 502 for D-Type 106, 1149 for A1–B1 107, 520 for C1 115, and 2010 for SMARTCITY LAB. No missing or duplicate gml:id values were identified within the supported metadata collections. Each supported metadata entity corresponds to one retrieval document, yielding the same total of 4181 documents. These counts describe the processed metadata and retrieval corpus, rather than geometry primitives or all CityGML feature types.

#### 4.1.2. Retrieval and Workflow Configuration

The evaluated configurations differ by execution path. BM25 retrieval uses an in-memory lexical index and does not require embeddings. Dense retrieval uses intfloat/multilingual-e5-base with the model revision specified in Section 3.3.3. The encoder produces 768-dimensional, L2-normalized vectors, and retrieval uses exact cosine similarity on the CPU. Both methods apply dataset filtering and return the ten highest-ranked documents. The previously configured Nomic embedding model was not evaluated.

The coordinated metadata workflow uses rule-based query analysis, direct metadata handlers, evidence validation, and bounded follow-up execution. It does not invoke a generative language model in the reported experiments. The default recovery limit permits at most two follow-up batches. The optional Ollama-backed summarization interface is described in Section 3.4.5; its model and decoding settings do not apply to the reported retrieval or coordination measurements.

#### 4.1.3. Software and Hardware Environment

The evaluated components were implemented in Python 3.11.13. The metadata workflow operates on model-specific canonical metadata artifacts using rule-based query analysis, deterministic coordination, and direct metadata handlers. Standalone retrieval uses an in-memory BM25 index or precomputed dense document vectors with exact cosine similarity. These execution paths are evaluated separately, as described in Section 4.2.

All experiments were conducted on a workstation equipped with an AMD Ryzen Threadripper PRO 5955WX processor, an NVIDIA GeForce RTX 3090 GPU with 24,095 MiB of video memory, and 32 GB of system memory. Both BM25 and Dense E5 retrieval were executed on the CPU. The available GPU does not indicate GPU use in the reported retrieval or metadata coordination measurements. Table 3 summarizes the software, hardware, and path-specific experimental configurations.

### 4.2. System Implementation and Query Processing Workflow

The implementation separates offline metadata preparation from online query execution. Two execution paths are evaluated: standalone metadata retrieval using BM25 or Dense E5, and the coordinated deterministic metadata workflow. Both use metadata derived from the four CityGML sources, but are evaluated separately because retrieval ranking and coordinated metadata processing perform different operations.

During offline preparation, a streaming parser extracts metadata from each source GML file and writes a model-specific canonical metadata artifact. Output validation, serialization, and hashing are recorded separately from parsing and extraction. Repeated runs are checked against version-locked reference artifacts to verify output consistency. The preprocessing measurements in Section 4.5 cover this metadata preparation path and exclude detailed geometry extraction and connectivity-graph construction.

For standalone retrieval, the extracted metadata is represented as a shared corpus of 4181 documents. BM25 uses an in-memory lexical index, whereas Dense E5 uses precomputed document embeddings and exact cosine similarity. Both methods restrict candidates to the selected dataset and return the ten highest-ranked documents. They use the same locked corpus, benchmark queries, and reference relevance sets, without score fusion. Retrieval effectiveness is reported in Section 4.3, and online latency is reported in Section 4.4. This path excludes agent coordination, geometric analysis, and LLM response generation.

**Figure 2:**
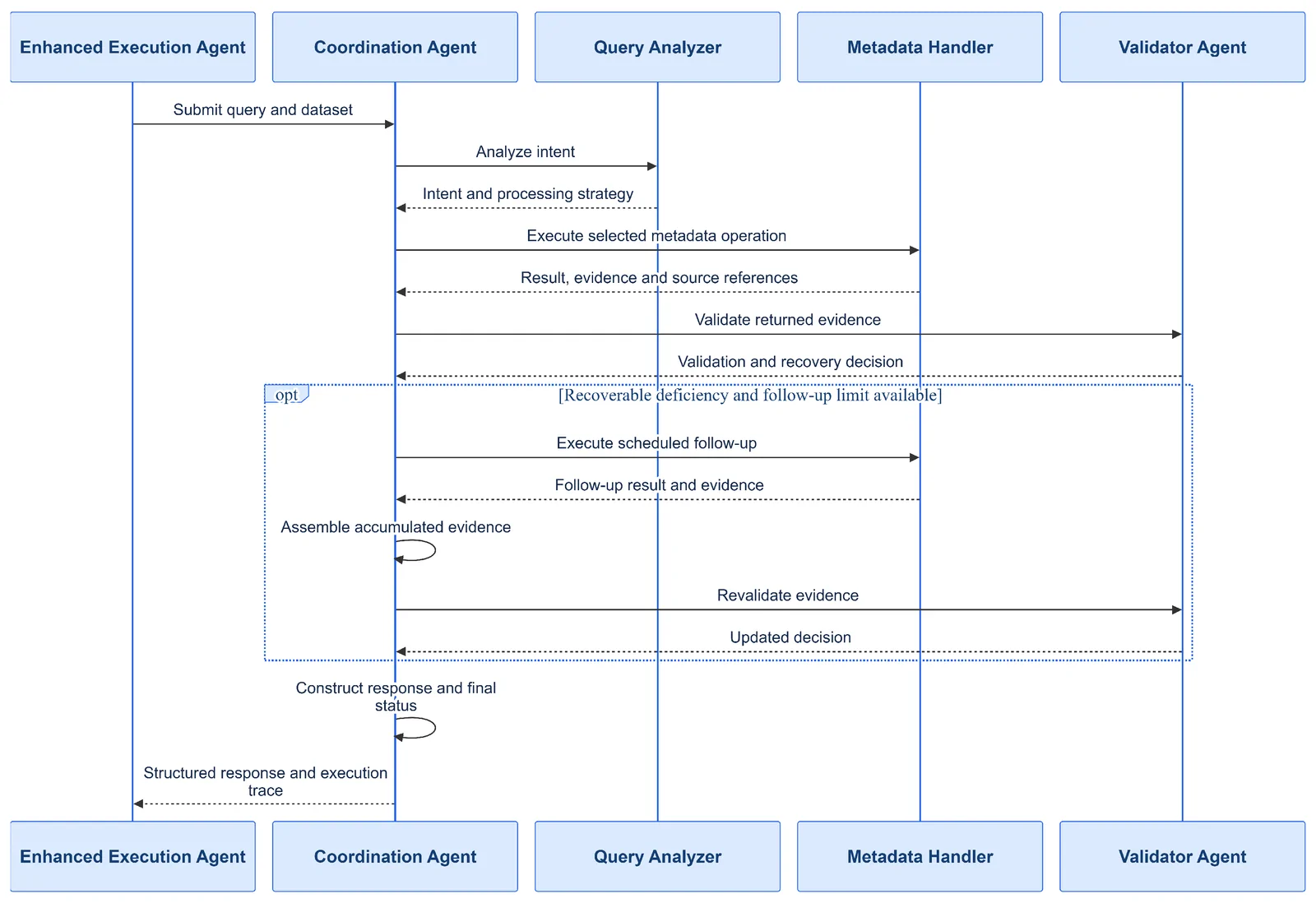
Sequence of interactions in the deterministic metadata workflow. The coordinator obtains the query intent, executes the selected metadata operation, and submits the returned evidence for validation. The optional fragment illustrates one follow-up batch; up to two batches are permitted while recovery remains possible and evidence has not been accepted. The coordinator returns a structured response, final status, and execution trace. Detailed termination conditions are specified in Algorithm 1. Solid arrows denote requests or processing calls, whereas dashed arrows denote returned results or responses. Self-directed arrows indicate internal processing within the same component.

The deterministic metadata workflow is evaluated separately. The selected model’s canonical metadata is loaded before query execution. As illustrated in Figure 2, EnhancedExecutionAgent forwards the query and selected dataset to CoordinationAgent, which requests rule-based intent analysis from the Query Analyzer. The returned intent and processing strategy determine the metadata handler to execute. The handler performs direct metadata processing and linear scans, returning results, evidence, and source references for validation by ValidatorAgent.

If validation identifies a recoverable deficiency and the configured follow-up limit has not been reached, the coordinator schedules additional execution, assembles the accumulated evidence, and requests revalidation. The optional fragment in Figure 2 illustrates one follow-up batch; the default configuration permits at most two batches. The coordinator then constructs a deterministic response with a final status and execution trace. Detailed termination conditions are specified in Algorithm 1. Figure 2 depicts only the coordinated metadata workflow; the separate BM25/dense retrieval path, geometric operations, and optional live LLM summarization are not shown.

Workflow instrumentation records agent activations, calls through the monitored vector-search boundary, LLM calls, and additional execution attempts. Activation counts include repeated validator method entries and therefore measure instrumented activity rather than the number of distinct agents. A same-operation retry is distinguished from a substantive revision of the execution plan. These definitions support interpretation of the coordination measurements; a zero count at the monitored vector-search boundary does not imply an absence of internal computation.

Measurement boundaries follow each evaluated path. Retrieval latency covers online execution after the required indexes and model have been initialized. For Dense E5, this includes query embedding and exact cosine search over precomputed document vectors. Offline preprocessing, document embedding, index construction, and model loading are excluded. Coordination measurements describe activity in the deterministic metadata workflow, including repeated attempts. Preprocessing time and memory are measured separately, while query-memory measurements include the loaded metadata and initialized workflow components. The shared BM25 index is profiled independently, and its memory is not allocated to individual model workflows. These boundaries distinguish retrieval, coordination, and resource measurements without treating them as end-to-end framework performance.

### 4.3. Retrieval Performance Across CityGML Models

The retrieval experiment evaluates the BM25 baseline on metadata derived from the four CityGML models. The retrieval corpus contains 4181 metadata documents, and the benchmark comprises 80 unique queries, with 20 queries associated with each model. This experiment measures the retrieval and ranking of relevant metadata documents. It does not evaluate generated answers, geometric reasoning, or the complete agent workflow.

#### 4.3.1. Benchmark Construction and Query Composition

The retrieval benchmark comprises 80 unique queries generated programmatically from canonical metadata, with 20 queries per CityGML dataset. Each dataset contributes five queries from four task groups: exact-identifier lookup, name lookup, attribute-value lookup, and entity-type-constrained attribute lookup. Table 4 groups the first two tasks as object queries and the latter two as attribute queries. Selection followed deterministic rules over entities ordered by type and gml:id: exact-identifier queries selected midpoint entities using a fixed type-priority order, while name queries selected the first named entity from five distinct types. Attribute-value queries used distinct non-identifier generic-attribute keys matching 2–100 entities; type-constrained queries used distinct type/key combinations matching 1–50 entities.

Each query has a binary relevance set containing all corpus entities satisfying its generating predicate. Exact-identifier queries identify the target entity, name queries include same-type entities sharing the selected name, and attribute queries include entities matching the requested value and any specified type. All reference document identifiers were verified to occur exactly once within the selected dataset. These reference sets assess document retrieval and ranking rather than generated-answer correctness. BM25 and Dense E5 use the same corpus, queries, and relevance sets, with dataset filtering applied before ranking.

The benchmark was constructed from the same canonical metadata used to build the retrieval corpus and therefore represents an internal, metadata-derived evaluation rather than an independently authored collection of user queries. No separate training or development split was used. Retrieval parameters were fixed before scoring, and no test-set tuning was performed. These construction choices should be considered when interpreting the results beyond the evaluated query templates.

#### 4.3.2. Retrieval Metrics

Let $G_{q}$ denote the nonempty reference set for query *q*, and let $L_{q}^{\left(k\right)}$ denote the set of document identifiers returned at the first *k* ranks. Retrieval coverage and precision are measured using Recall@5 and Precision@5:

(4) $$\mathrm{Recall}@5_{q}=\frac{|L_{q}^{\left(5\right)}\cap G_{q}|}{|G_{q}|},\;\mathrm{Precision}@5_{q}=\frac{|L_{q}^{\left(5\right)}\cap G_{q}|}{5}.$$

The precision denominator remains five when fewer than five documents are returned.

Ranking quality is measured using MRR@10 and nDCG@10. The reciprocal rank for an individual query is

(5) $$\mathrm{RR}@10_{q}=\left\{\begin{array}{cc} 1/r_{q}, & \mathrm{if}\;\mathrm{the}\;\mathrm{first}\;\mathrm{relevant}\;\mathrm{result}\;\mathrm{occurs}\;\mathrm{at}\;r_{q}\leq 10,\\ 0, & \mathrm{if}\;\mathrm{no}\;\mathrm{relevant}\;\mathrm{result}\;\mathrm{occurs}\;\mathrm{in}\;\mathrm{the}\;\mathrm{first}\;\mathrm{ten}\;\mathrm{ranks}. \end{array}\right.$$

MRR@10 is the mean of these reciprocal ranks over the evaluated queries.

For nDCG@10, binary relevance is assigned to each returned document. Let ${\mathrm{rel}}_{q,i}=1$ when the document at rank *i* belongs to $G_{q}$, and zero otherwise. Then

(6) $$\mathrm{DCG}@10_{q}=\sum_{i=1}^{10}\frac{{\mathrm{rel}}_{q,i}}{\log_{2}\left(i+1\right)},\;\mathrm{nDCG}@10_{q}=\frac{\mathrm{DCG}@10_{q}}{\mathrm{IDCG}@10_{q}},$$

where $\mathrm{IDCG}@10_{q}$ is the DCG of an ideal ranking with relevant documents placed first. Missing result positions contribute zero.

Retrieval performance was further analyzed by query type and reference-set size using the saved rankings from the 80-query benchmark. The four query types each contained 20 queries, while reference sets were grouped into sizes of 1, 2–5, 6–10, and more than 10 relevant documents. Each query contributed one quality observation per method; latency repetitions were excluded from this analysis.

For a query *q* with reference set $G_{q}$, the maximum attainable Recall@5 was defined as

$$C_{q}@5=\frac{\min(5,|G_{q}|)}{|G_{q}|}.$$

To distinguish retrieval performance from the fixed-cutoff constraint, normalized Recall@5 was calculated as

$${\mathrm{NR}}_{q}@5=\frac{{\mathrm{Recall}}_{q}@5}{C_{q}@5}.$$

Group-level values were obtained by averaging the query-level metrics and ceilings.

#### 4.3.3. Cross-Model Results

Table 5 compares BM25 and dense E5 retrieval across the four CityGML models. BM25 achieved higher scores on all four metrics for every model. Overall, BM25 obtained Recall@5 of 0.5325, Precision@5 of 0.5225, MRR@10 of 0.9150, and nDCG@10 of 0.8994. The corresponding dense retrieval scores were 0.1273, 0.1975, 0.2740, and 0.2722, respectively. These results favor BM25 under the evaluated metadata representation and query distribution; they do not establish that lexical retrieval is generally superior to dense retrieval for CityGML queries.

For BM25, Recall@5 varied narrowly between 0.5279 and 0.5352, whereas MRR@10 ranged from 0.8667 to 0.9600. D-Type 106 achieved the highest MRR@10 and nDCG@10, while SMARTCITY LAB achieved the highest Recall@5 and Precision@5. Dense retrieval showed greater variation across models. D-Type 106 obtained its highest Recall@5 at 0.1652, whereas C1 115 obtained its highest Precision@5, MRR@10, and nDCG@10 at 0.3800, 0.4542, and 0.4597, respectively. A1–B1 107 produced the lowest scores for both methods.

The relatively high BM25 ranking scores indicate that relevant documents were frequently placed near the top of the retrieved list. However, its overall Recall@5 remained approximately 0.53. Ranking quality therefore should not be interpreted as complete evidence coverage: when a query has more than five relevant documents, complete recall is unattainable at this cutoff even with an ideal ranking. Document-level relevance also does not establish the correctness of extracted attributes or aggregated answers.

These findings are limited to an internal benchmark of 80 metadata-derived queries whose reference relevance sets were programmatically cross-checked but had not undergone independent human review. The dense condition also applied a 512-token encoder limit in addition to the shared 4096-character document cap, potentially excluding information available to BM25. The observed differences should therefore be interpreted within this configuration. Neither the cross-model comparison nor the source polygon counts establish geometric reasoning performance or city-scale scalability.

Statistical analysis treats each unique query as one observation as shown in Table 6, pairing BM25 and Dense E5 results by dataset and query identifier. We computed 95% percentile-bootstrap confidence intervals using 10,000 replicates, sampling 20 query pairs with replacement within each dataset. Differences between methods were assessed using two-sided paired Monte Carlo permutation tests with 100,000 permutations and the absolute mean paired difference as the test statistic. Monte Carlo *p*-values were computed as (b+1)/(B+1), where *b* counts permutation statistics at least as large as the observed statistic. Holm correction was applied across the four metrics. The random seed was 42. Latency repetitions were not treated as independent retrieval-quality observations.

All four paired comparisons favored BM25. None of the 100,000 sampled permutations produced an absolute mean difference at least as large as the observed difference for any metric. Using the stated Monte Carlo correction, each test yielded $p_{\mathrm{raw}}\approx 1.00\times 10^{-5}$, with $p_{\mathrm{Holm}}\approx 4.00\times 10^{-5}$ after adjustment across the four metrics. These results indicate statistically significant differences within the evaluated benchmark, consistent with the paired-difference confidence intervals reported in Table 6.

#### 4.3.4. Retrieval Performance by Query Type and Reference-Set Size

Table 7 reports retrieval performance by query type and reference-set size. BM25 achieved Recall@5 of 1.0000 for both exact-identifier and name queries, whereas Dense E5 achieved 0.0000 and 0.4500, respectively. For attribute-only and type-constrained attribute queries, BM25 achieved normalized Recall@5 of 0.7700 and 0.8900, compared with 0.1800 and 0.4900 for Dense E5. These results indicate that BM25 was more effective for the evaluated metadata queries, particularly those requiring exact identifier matching. This finding is specific to the benchmark templates and document representations used here.

Reference-set size substantially affected the interpretation of Recall@5. Forty queries had more than 10 relevant documents, with a mean attainable Recall@5 ceiling of 0.0765. Within this group, BM25 achieved Recall@5 of 0.0650 and normalized Recall@5 of 0.8300, whereas Dense E5 achieved 0.0296 and 0.3350, respectively. Low absolute recall in this group therefore reflects both the fixed retrieval cutoff and the ranking of relevant documents. The remaining queries comprised 39 singleton reference sets and one reference set containing 2–5 documents; no queries fell within the 6–10 group. Results for these groups should consequently be interpreted in view of their unequal sample sizes.

Document truncation was examined at the shared 4096-character corpus limit and the subsequent 512-token limit applied to E5. The character limit affected 1133 of 4181 corpus documents, while 4145 documents exceeded the E5 token limit after character truncation. Across 2965 query–relevant-document pairs, the reconstructed query-defining identifier, name, or attribute expression, was absent from the retained text in 513 pairs after character truncation and in a further 1439 pairs after E5 token truncation. These observations identify limitations in the retained input, but do not establish that truncation caused retrieval errors, since retrieval with untruncated inputs was not evaluated.

### 4.4. Latency and Agent Coordination Across Models

This subsection reports standalone retrieval latency for BM25 and Dense E5, execution activity of the deterministic metadata workflow, and correctness and latency comparisons with direct metadata processing and a workflow variant without validation and recovery.

#### 4.4.1. BM25 and Dense Retrieval Latency

Retrieval latency was measured for BM25 and Dense E5 using the same 80-query benchmark. Each query was executed over three measured repetitions after one excluded warm-up call per method, yielding 240 measured calls per method and 480 calls overall. Both methods were executed on the CPU. The reported median summarizes the per-query median latencies, whereas the 95th percentile is computed over individual measured calls.

The timing boundary covers online retrieval with the required indexes and model already initialized. For dense retrieval, this includes query embedding and exact cosine search over precomputed document vectors. Source preprocessing, document embedding, index construction, model loading, agent coordination, geometric operations, and LLM response generation are excluded.

Table 8 reports an overall median latency of 4.80 ms for BM25 and 68.10 ms for Dense E5, with corresponding p95 values of 14.51 ms and 81.71 ms. Across datasets, BM25 median latency ranged from 4.14 ms for C1 115 to 6.07 ms for A1–B1 107. Dense retrieval showed the same minimum and maximum datasets, with median latencies of 66.13 ms and 69.75 ms, respectively. The highest p95 latency was 15.76 ms for BM25 on SMARTCITY LAB and 83.24 ms for dense retrieval on C1 115.

Dense retrieval includes online query embedding, whereas BM25 operates directly on the initialized lexical index. These measurements describe retrieval latency under the evaluated CPU configuration and exclude offline preparation, agent coordination, geometric operations, and LLM generation. They therefore do not represent end-to-end framework response times. Retrieval effectiveness is assessed separately in Table 5.

#### 4.4.2. Deterministic Metadata Workflow Coordination

The coordination experiment comprises 32 unique metadata queries, with eight queries per model and three repetitions per query, yielding 96 executions. This workload is separate from the 80-query BM25/dense retrieval benchmark. The workflow uses deterministic query analysis, direct metadata handlers, evidence validation, and a recovery limit of two follow-up batches. Optional live LLM summarization was disabled.

Each dataset contributed six recurring tasks: listing Room identifiers, counting Rooms, retrieving an existing Room, retrieving Room attributes, requesting a missing Room identifier, and requesting an existing non-Room identifier as a Room. Two additional exact-identifier queries per dataset were drawn from the retrieval benchmark, yielding eight workflow queries per dataset. The two benchmarks evaluate different processing paths, although some target identifiers overlap.

To assess answer correctness separately from execution activity, expected answers were reconstructed using an independent streaming XML procedure over the four source CityGML files. This procedure did not import the production query analyzer, metadata handlers, coordinator, validator, or answer builder. Room listings were compared using set equality over exact gml:id values, and room counts included uniquely identified bldg:Room elements. Entity lookups checked both the requested identifier and entity type. Names and generic attributes were whitespace-trimmed but otherwise compared exactly. Source absence was distinguished from extraction failure. The resulting reference answers and source evidence were checked automatically; independent human verification remains pending.

Each execution’s structured result, final answer text, and validation status were assessed separately. Outcomes were counted once per unique query, while the three repetitions were used to assess consistency. Under the evidence-based scoring rule described in Section 4.4.3, the full workflow produced 22 correct answers, four appropriate abstentions, four incorrect answers, and two failures to answer. All outcomes were consistent across repetitions. The four incorrect answers concerned identifier/type-mismatch requests: existing Door identifiers were described as unavailable rather than identified as belonging to a different entity type. Although structured results matched the references for all 32 queries, two valid handler outputs were rejected during validation. Ten queries therefore ended with an insufficient-evidence status, but only four represented appropriate abstentions.

Table 9a reports workflow execution activity, while Table 9b summarizes the audited answer outcomes. Mean activation counts were 4.00 for D-Type 106 and C1 115, and 4.50 for A1–B1 107 and SMARTCITY LAB. These counts include repeated validator method entries and therefore do not represent the number of distinct agents. The two additional-activity cases each executed two same-operation retries. No substantive plan revision, call through the monitored vector-search boundary, or live LLM call was observed. These zero call counts apply to the coordinated metadata path and do not describe the separate dense retrieval experiment.

The two failures to answer occurred in A1–B1 107 and SMARTCITY LAB. The Door entities were present and correctly returned by the metadata handler, but the validator incorrectly rejected the available evidence and produced insufficient-evidence responses. Each case triggered two follow-up verification attempts, but the framework obtained no additional evidence and the validation failure remained unresolved. These retries therefore did not improve the final answers, exposing a limitation of the current metadata-only recovery mechanism.

#### 4.4.3. Comparison with Direct Metadata Processing and Workflow Ablation

Three configurations were compared on the same 32 metadata queries: direct metadata processing, the workflow without validation and recovery, and the full workflow. Direct processing invoked the existing metadata handlers through a fixed query-to-handler and parameter mapping, assuming known query interpretation. It bypassed query analysis, coordination, validation, and recovery. The ablated workflow retained query analysis, coordination, and metadata handlers but returned the initial result without validation or follow-up. The full workflow retained the implemented validation procedure and allowed at most two follow-up batches.

Metadata loading and component initialization were completed before timing. One warm-up call per dataset and configuration was excluded, final-answer caching was disabled, and each query was measured three times, yielding 96 measured calls per configuration. Online latency covered query receipt through response return, including validation and recovery where enabled. Median latency summarizes the 32 per-query medians, whereas p95 is calculated over the 96 measured calls. Dataset selection, initialization, warm-up, and answer scoring were excluded from the timing boundary.

Answers were assessed against references reconstructed independently from the CityGML files. Positive responses were checked using the exact-content rules described in Section 4.4.2. Negative responses were assessed according to their meaning, distinguishing an absent identifier from an identifier associated with a different entity type. This evidence-based rule replaced the initial phrase-based recognition of abstentions and was applied to all 288 recorded executions. Original responses and latency measurements were preserved. Independent human verification of the references remains pending.

Table 10 summarizes the results. Direct processing and the ablated workflow each produced 24 correct positive answers and eight appropriate negative responses, with no incorrect answers or failures to answer. The full workflow produced 22 correct answers, four appropriate negative responses, four incorrect answers, and two failures to answer. The four incorrect responses described existing Door identifiers as unavailable instead of explaining that they did not match the requested Room type. The two failures to answer resulted from false validator rejection of valid handler outputs, as discussed in Section 4.4.2.

Structured results matched the references for all 32 queries in every configuration, and all outcomes were consistent across repetitions. No execution errors were recorded. Thus, the differences in final-answer assessment arose after metadata processing. Counting correct positive answers and appropriate negative responses, direct processing and the ablated workflow achieved 32 correct outcomes, compared with 26 for the full workflow.

Median latency was 0.027 ms for direct processing, 3.770 ms for the ablated workflow, and 36.842 ms for the full workflow; corresponding p95 values were 0.174, 22.991, and 294.729 ms. The full workflow therefore incurred additional execution cost without improving final-answer correctness on this workload. The combined ablation does not isolate validation cost from recovery cost. Moreover, the direct baseline assumes known query interpretation, and the results are limited to the internal 32-query metadata benchmark. They identify limitations in validation and response construction without establishing the relative performance of these configurations on broader tasks.

### 4.5. Preprocessing and Resource Consumption

This subsection characterizes the offline preparation costs and resource requirements of the four CityGML models. Preprocessing is assessed separately from the online query latency reported in Section 4.4. The analysis distinguishes source parsing, feature extraction, index construction, and output serialization so that the cost of preparing a model is not conflated with the cost of answering individual queries.

#### 4.5.1. Preprocessing Time

Canonical metadata preprocessing was measured over three repetitions per CityGML model, with each repetition executed in a fresh process and a separate output directory. Each run reparsed the raw GML file rather than reusing previously generated metadata. Source parsing and metadata extraction were timed together because the streaming parser interleaves these operations. Output validation, JSON serialization, hashing, and total wall time were recorded separately. The generated metadata hashes matched the locked Phase 2 artifacts in all measured runs.

Table 11 reports mean processing times and standard deviations. Mean parse/extract time was 1.088 s for D-Type 106, 2.827 s for A1–B1 107, 1.004 s for C1 115, and 13.750 s for SMARTCITY LAB. Metadata serialization required between 0.380 s and 0.972 s. Total preprocessing wall time, including validation, serialization, hashing, and associated overhead, was 1.536±0.016 s, 3.947±0.168 s, 1.471±0.015 s, and 15.151±0.127 s, respectively. These measurements characterize metadata-only processing and exclude geometry extraction.

The evaluated production metadata handlers use linear scans rather than a separately constructed lookup index. Lookup-index and connectivity-graph construction are therefore marked *NI* in Table 11. BM25 indexing was measured separately over the shared corpus of 4181 metadata documents. In the additional resource-measurement run, index construction took 1.2205 s. This shared cost is not allocated to individual models and is excluded from their preprocessing times. Dense-vector and spatial-index construction were not evaluated in these runs.

#### 4.5.2. Memory and Storage Requirements

Process resident set size (RSS) was sampled at 10 ms intervals using Windows GetProcessMemoryInfo. Each preprocessing repetition ran in a fresh process. Table 12 reports the maximum sampled preprocessing peak across three repetitions per model. Query-memory measurements were collected in a separate fresh worker for each dataset, beginning after metadata loading and agent initialization. The reported query RAM is the absolute peak RSS during the deterministic metadata query sequence, including the loaded data and initialized components. Sampling may miss peaks shorter than the sampling interval.

Storage measurements distinguish the raw GML files from the canonical metadata artifacts, using $1\;\mathrm{MiB}=2^{20}$ bytes. Raw file sizes ranged from 24.97 MiB to 372.03 MiB, whereas metadata sizes ranged from 11.80 MiB to 28.18 MiB. Metadata sizes exclude geometry, manifests, and retrieval-corpus files. Despite having the largest raw file, SMARTCITY LAB did not have the largest metadata artifact or preprocessing memory peak. This observation indicates that raw file size alone does not determine the resource requirements of metadata-only processing.

The BM25 index was evaluated separately as a shared in-memory index over 4181 documents. Its construction run reached a sampled process peak of 244.32 MiB, with a before-to-after RSS increase of 202.70 MiB. This increase represents the process footprint change during index construction, rather than an isolated measurement of index object size. No serialized index artifact was produced; the IM entries in Table 12 therefore indicate in-memory storage and are not zero-size measurements. Shared-index memory is not allocated to individual datasets and is excluded from the production query-memory values.

## 5. Discussion

The evaluation characterizes metadata retrieval and deterministic query coordination over four static CityGML 2.0 building models. BM25 achieved overall Precision@5, Recall@5, and MRR@10 values of 0.5225, 0.5325, and 0.9150, respectively, outperforming dense E5 under the evaluated representation and query templates. Published CityGML interfaces assess related tasks through answer-level or query-output evaluations [16,46]. Their results cannot be directly ranked against the document-level retrieval metrics reported here because query workloads, reference construction, and processing boundaries differ. The matched BM25–E5 comparison therefore supports conclusions within the present benchmark, rather than superiority over published systems.

Reference-set size and document truncation qualify the interpretation of retrieval performance. For the 40 queries with more than 10 relevant documents, the mean Recall@5 ceiling was 0.0765, while normalized Recall@5 was 0.8300 for BM25 and 0.3350 for dense E5. Low absolute recall therefore reflects both ranking performance and the fixed retrieval cutoff. The shared 4096-character limit and the additional E5 512-token limit also removed query-defining information from some inputs. However, their effect on ranking cannot be isolated without comparison against untruncated retrieval.

The workflow comparison identifies limitations in validation and response construction. Although structured results matched independently reconstructed references for all 32 queries, direct processing and the workflow without validation and recovery each produced 32 correct final outcomes, compared with 26 for the full workflow. The latter produced four incorrect responses to entity-type mismatches and two failures caused by rejection of valid evidence. Follow-up attempts obtained no additional evidence and did not resolve the two failures. These results show that internal validation status and bounded retries do not by themselves establish final-answer correctness.

The timing results indicate the cost of the evaluated processing stages. Median standalone retrieval latency was 4.80 ms for BM25 and 68.10 ms for dense E5, including online query embedding for E5. On the separate workflow benchmark, median latency was 0.027 ms for direct processing, 3.770 ms without validation and recovery, and 36.842 ms for the full workflow. The additional cost did not improve correctness on this workload, although direct processing assumed known query interpretation and the ablation did not isolate validation from recovery. Metadata preprocessing required approximately 1.47–15.15 s and produced 11.80–28.18 MiB artifacts; these measurements exclude detailed geometry processing.

Generalization remains limited by the four-building collection, programmatically constructed queries, pending human verification, and restricted access to the underlying models. The candidate connectivity graph and Dijkstra routing implementation require independent spatial validation, while optional live LLM summarization was not evaluated. Future work should prioritize evidence-preserving document representations, validation and response rules, and independently verified spatial relationships before evaluating language-model generation and dynamic sensor integration. The current framework is therefore best interpreted as a potential query layer over static building information.

## 6. Conclusions

This study presented an agent-oriented framework for querying static CityGML building models through structured metadata, deterministic query coordination, evidence validation, and bounded recovery. Evaluation used four CityGML 2.0 models containing 4181 processed metadata entities, with separate benchmarks of 80 retrieval queries and 32 workflow queries. The experiments assessed retrieval quality, final-answer correctness, online latency, and metadata preprocessing requirements.

BM25 outperformed dense E5 on the evaluated retrieval benchmark, achieving overall MRR@10 of 0.9150 and median retrieval latency of 4.80 ms. Analysis by query type and reference-set size showed that retrieval coverage depends on both ranking performance and the fixed cutoff. In the workflow comparison, direct processing and the variant without validation and recovery each produced 32 correct final outcomes, compared with 26 for the full workflow. The additional validation and recovery increased latency without improving correctness on this workload, identifying limitations in evidence validation and response construction.

These findings are limited to static building metadata and the evaluated query templates. Future work will prioritize preserving query-relevant information, correcting validation and response rules, and independently verifying reference answers and spatial relationships. Live LLM generation, routing over validated connectivity, and sensor-based state updates require further evaluation before the framework can be assessed as a query component for dynamically synchronized digital twins.

## Figures and Tables

**Table 1:** Source inventory of four CityGML models.

Model	Rooms	Doors	Windows	Stairs	Runs	Polygons
D-Type 106	16	12	18	7	8	34,481
A1–B1 107	21	26	33	11	18	93,054
C1 115	19	11	16	8	8	30,195
SMARTCITY LAB	18	33	28	0	4	714,083

**Table 2:** Processed metadata entities and retrieval documents for the four CityGML datasets. Each supported metadata entity corresponds to one retrieval document. Counts exclude geometry primitives and unsupported feature types.

Model	Processed MetadataEntities	RetrievalDocuments
D-Type 106	502	502
A1–B1 107	1149	1149
C1 115	520	520
SMARTCITY LAB	2010	2010
Total	4181	4181

**Table 3:** Prototype software, hardware, and model configurations. LLM settings apply to optional summarization, which was not exercised in the reported experiments. Dense E5 was evaluated through the standalone retrieval path.

Configuration Item	Configuration Setting
Optional LLM summarization	
LLM and model tag	gpt-oss:20b
Model quantization	MXFP4
Inference runtime	Ollama, version 0.32.0
Configured context window	131,072 tokens
Temperature	0.1
Top-*p*	0.9
Maximum output tokens	2000
Random seed	42
Dense retrieval (evaluated)	
Embedding model	intfloat/multilingual-e5-base (768-dimensional embeddings)
Software and hardware	
Orchestration framework	LangChain 0.0.340
Programming environment	Python 3.11.13
CPU	AMD Ryzen Threadripper PRO 5955WX
GPU and VRAM	NVIDIA GeForce RTX 3090, 24,095 MiB
System memory	32 GB

**Table 4:** Metadata retrieval queries by dataset and task group.

Model	Object	Attribute	Total
D-Type 106	10	10	20
A1–B1 107	10	10	20
C1 115	10	10	20
SMARTCITY LAB	10	10	20
Total	40	40	80

**Table 5:** BM25 and dense retrieval performance on the internal metadata benchmark. Each dataset contributes 20 unique queries; overall values are macro-averaged over 80 queries.

Model	Method	Recall@5	Precision@5	MRR@10	nDCG@10
D-Type 106	BM25	0.5336	0.5500	0.9600	0.9407
	Dense E5	0.1652	0.2100	0.3000	0.2913
A1–B1 107	BM25	0.5279	0.4700	0.8667	0.8442
	Dense E5	0.1011	0.0400	0.1000	0.1008
C1 115	BM25	0.5334	0.5100	0.9500	0.9175
	Dense E5	0.1288	0.3800	0.4542	0.4597
SMARTCITY LAB	BM25	0.5352	0.5600	0.8833	0.8951
	Dense E5	0.1141	0.1600	0.2417	0.2369
Overall	BM25	0.5325	0.5225	0.9150	0.8994
	Dense E5	0.1273	0.1975	0.2740	0.2722

**Table 6:** Statistical comparison of BM25 and Dense E5 on 80 paired queries. Means and 95% confidence intervals use 10,000 dataset-stratified paired bootstrap samples; differences are BM25 minus Dense E5. Two-sided paired Monte Carlo permutation tests use 100,000 permutations. $p_{\mathrm{raw}}$ and $p_{\mathrm{Holm}}$ denote unadjusted and Holm-adjusted *p*-values across four metrics, respectively.

Metric	Statistic	BM25	Dense E5	Difference
Recall@5	Mean	0.5325	0.1273	0.4052
	95% CI	[0.4295, 0.6361]	[0.0641, 0.2005]	[0.3054, 0.5129]
	$p_{\mathrm{raw}}\approx 1.00\times 10^{-5}$; $p_{\mathrm{Holm}}\approx 4.00\times 10^{-5}$			
Precision@5	Mean	0.5225	0.1975	0.3250
	95% CI	[0.4375, 0.6075]	[0.1325, 0.2650]	[0.2475, 0.4050]
	$p_{\mathrm{raw}}\approx 1.00\times 10^{-5}$; $p_{\mathrm{Holm}}\approx 4.00\times 10^{-5}$			
MRR@10	Mean	0.9150	0.2740	0.6410
	95% CI	[0.8571, 0.9646]	[0.1906, 0.3594]	[0.5490, 0.7327]
	$p_{\mathrm{raw}}\approx 1.00\times 10^{-5}$; $p_{\mathrm{Holm}}\approx 4.00\times 10^{-5}$			
nDCG@10	Mean	0.8994	0.2722	0.6272
	95% CI	[0.8419, 0.9483]	[0.1903, 0.3574]	[0.5339, 0.7196]
	$p_{\mathrm{raw}}\approx 1.00\times 10^{-5}$; $p_{\mathrm{Holm}}\approx 4.00\times 10^{-5}$			

**Table 7:** Retrieval performance by query type and reference-set size across the 80-query benchmark.

Group	Method	*n*	P@5	R@5	MRR@10	Ceiling@5	Norm. R@5
*(a) Query type*							
Exact identifier	BM25	20	0.2000	1.0000	0.9083	1.0000	1.0000
	Dense E5	20	0.0000	0.0000	0.0000	1.0000	0.0000
Name	BM25	20	0.2300	1.0000	0.9667	1.0000	1.0000
	Dense E5	20	0.1200	0.4500	0.4250	1.0000	0.4500
Attribute	BM25	20	0.7700	0.0398	0.8600	0.0517	0.7700
	Dense E5	20	0.1800	0.0094	0.1458	0.0517	0.1800
Type and attribute	BM25	20	0.8900	0.0902	0.9250	0.1012	0.8900
	Dense E5	20	0.4900	0.0498	0.5250	0.1012	0.4900
*(b) Reference-set size, $|G_{q}|$*							
1	BM25	39	0.2000	1.0000	0.9359	1.0000	1.0000
	Dense E5	39	0.0410	0.2051	0.1923	1.0000	0.2051
2–5	BM25	1	0.8000	1.0000	1.0000	1.0000	1.0000
	Dense E5	1	0.8000	1.0000	1.0000	1.0000	1.0000
6–10	BM25	0	–	–	–	–	–
	Dense E5	0	–	–	–	–	–
>10	BM25	40	0.8300	0.0650	0.8925	0.0765	0.8300
	Dense E5	40	0.3350	0.0296	0.3354	0.0765	0.3350

**Table 8:** CPU latency of the standalone BM25 and Dense E5 retrieval paths on 80 unique queries, with three measured repetitions per query and one excluded warm-up call per method. Each method contributes 60 measured calls per dataset. Median values summarize per-query median latencies; p95 values summarize individual measured calls. Dense latency includes query embedding and vector search. Measurements exclude offline preparation, model loading, agent coordination, geometric operations, and LLM generation; they are not end-to-end framework latencies.

Model	BM25Median (ms)	BM25p95 (ms)	DenseMedian (ms)	Densep95 (ms)
D-Type 106	5.19	14.57	68.93	80.65
A1–B1 107	6.07	14.25	69.75	81.81
C1 115	4.14	13.63	66.13	83.24
SMARTCITY LAB	4.25	15.76	66.92	78.11
Overall	4.80	14.51	68.10	81.71

**Table 9:** Execution activity and audited answer outcomes of the deterministic metadata workflow on 32 unique queries (eight per dataset), with three repetitions per query. Activity counts are averaged across repetitions and queries; retry and revised-plan rates use unique queries. Answer outcomes are counted once per unique query against references reconstructed independently from source CityGML. All outcomes were consistent across repetitions. Independent human verification remains pending.

*(a) Workflow execution activity*					
Model	Activations	Vector-search calls	LLM calls	Retry (%)	Revised plan (%)
D-Type 106	4.00	0.00	0.00	0.00	0.00
A1–B1 107	4.50	0.00	0.00	12.50	0.00
C1 115	4.00	0.00	0.00	0.00	0.00
SMARTCITY LAB	4.50	0.00	0.00	12.50	0.00
Overall	4.25	0.00	0.00	6.25	0.00
*(b) Workflow Answer Assessment*					
Model	Queries	Correct answer	Appropriate abstention	Failure to answer	Incorrect answer
D-Type 106	8	6	1	0	1
A1–B1 107	8	5	1	1	1
C1 115	8	6	1	0	1
SMARTCITY LAB	8	5	1	1	1
Overall	32	22	4	2	4

**Table 10:** Final-answer outcomes and online latency for three configurations evaluated on the same 32 metadata queries. Outcome counts use unique queries; each configuration includes 96 measured calls. Median latency summarizes per-query medians, while p95 summarizes individual calls. Negative responses are assessed against CityGML evidence, distinguishing missing identifiers from entity-type mismatches. Independent human verification remains pending.

Configuration	CorrectPositive	AppropriateNegative	IncorrectAnswer	Failureto Answer	Median(ms)	p95(ms)
Direct metadata processing	24	8	0	0	0.027	0.174
Workflow without validation/recovery	24	8	0	0	3.770	22.991
Full workflow	22	4	4	2	36.842	294.729

**Table 11:** Canonical metadata preprocessing time by CityGML model. Values are mean ± standard deviation in seconds over three fresh-process runs. *NI* indicates a stage not implemented in the evaluated metadata pipeline. Parse/extract excludes output serialization and hashing.

Model	Parse/Extract	Lookup Index	Graph	Serialize
D-Type 106	1.088±0.013	*NI*	*NI*	0.380±0.017
A1–B1 107	2.827±0.062	*NI*	*NI*	0.972±0.092
C1 115	1.004±0.006	*NI*	*NI*	0.386±0.005
SMARTCITY LAB	13.750±0.123	*NI*	*NI*	0.956±0.003

**Table 12:** Measured storage and process memory by CityGML model. All numeric values are in MiB ($1\;\mathrm{MiB}=2^{20}$ bytes). Metadata excludes geometry and manifest files. Peak RAM is the maximum sampled preprocessing RSS across three runs; query RAM is the sampled peak RSS during the production query sequence after setup. IM denotes a shared in-memory BM25 index without a serialized index artifact.

Model	Source	Processed	Index	Peak RAM	Query RAM
D-Type 106	27.09	11.80	IM	123.24	75.92
A1–B1 107	73.05	28.18	IM	249.68	110.65
C1 115	24.97	11.99	IM	124.35	76.38
SMARTCITY LAB	372.03	26.23	IM	241.85	106.25

## Data Availability

The CityGML datasets used in this study are subject to third-party access and redistribution restrictions and are not publicly available. Requests for access may be directed to the corresponding author and are subject to permission from the data owners. The implementation code is available from the corresponding author upon reasonable request.

## References

[1] Umeike R., Dao T., Crawford S. (2025). State-of-the-art review of resilient smart cities: Progress and challenges. Urban Gov..

[2] Bittencourt J.C.N., Costa D.G., Portugal P., Vasques F. (2024). A Survey on Adaptive Smart Urban Systems. IEEE Access.

[3] Adeleke O.J., Jovanovich K.D., Ogunbunmi S., Samuel O., Kehinde T.O. (2025). Comprehensive Exploration of Smart Cities: A Systematic Review of Benefits, Challenges, and Future Directions in Telecommunications and Urban Development. IEEE Sens. Rev..

[4] Huzzat A., Anpalagan A., Khwaja A.S., Woungang I., Alnoman A.A., Pillai A.S. (2025). A comprehensive review of Digital Twin technologies in smart cities. Digit. Eng..

[5] Mylonas G., Kalogeras A., Kalogeras G., Anagnostopoulos C., Alexakos C., Muñoz L. (2021). Digital Twins From Smart Manufacturing to Smart Cities: A Survey. IEEE Access.

[6] (2012). OGC City Geography Markup Language (CityGML): Encoding Standard.

[7] Gröger G., Plümer L. (2012). CityGML – Interoperable semantic 3D city models. ISPRS J. Photogramm. Remote Sens..

[8] Prandi F., De Amicis R., Piffer S., Soave M., Cadzow S., Gonzalez Boix E., D’Hont E. (2013). Using CityGML to Deploy Smart-City Services for Urban Ecosystems. Int. Arch. Photogramm. Remote Sens. Spat. Inf. Sci..

[9] Noardo F., Arroyo Ohori K., Biljecki F., Ellul C., Harrie L., Krijnen T., Eriksson H., van Liempt J., Pla M., Ruiz A. (2020). Reference study of CityGML software support: The GeoBIM benchmark 2019—Part II. Trans. GIS.

[10] Tan Y., Liang Y., Zhu J. (2023). CityGML in the Integration of BIM and the GIS: Challenges and Opportunities. Buildings.

[11] Braun R., Weiler V., Zirak M., Dobisch L., Coors V., Eicker U. Using 3D CityGML Models for Building Simulation Applications at District Level. Proceedings of the 2018 IEEE International Conference on Engineering, Technology and Innovation (ICE/ITMC).

[12] Hagos D.H., Battle R., Rawat D.B. (2024). Recent Advances in Generative AI and Large Language Models: Current Status, Challenges, and Perspectives. IEEE Trans. Artif. Intell..

[13] Pan F., Huang X., Bi Y., Gao Y., Ye Y., Wang H. (2025). From tools to partners: How large language models are transforming urban planning. AI Open.

[14] Hindi M., Mohammed L., Maaz O., Alwarafy A. (2025). Enhancing the Precision and Interpretability of Retrieval-Augmented Generation (RAG) in Legal Technology: A Survey. IEEE Access.

[15] Campo D.N., Conde J., Alonso Á., Huecas G., Salvachúa J., Reviriego P. (2025). Real-time Spatial Retrieval Augmented Generation for Urban Environments. arXiv.

[16] Liu S., Wang C. (2025). KCitychatBot: A knowledge graph based chatbot system for large-scale CityGML dataset. Int. Arch. Photogramm. Remote Sens. Spat. Inf. Sci..

[17] Kanna K., Kolbe T.H. (2025). Automatic Enrichment of Semantic 3D City Models using Large Language Models. ISPRS Ann. Photogramm. Remote Sens. Spat. Inf. Sci..

[18] Wei Z., Li X., He Z. (2022). Semantic Urban Vegetation Modelling Based on an Extended CityGML Description. J. Digit. Landsc. Archit..

[19] Pan Y., Hou M., Yang S., Li H., Wei S., Fan H. (2025). A CityGML ADE for modeling Buddhist statues in 3D with semantic information. J. Cult. Herit..

[20] Lasorella M., Cantatore E. (2025). CityGML-based model for the recovery and management of built cultural heritage: A systematic review towards digitalized processes. City Territ. Archit..

[21] Yang S., Chen J., Hou M., Fan H., Li S., Sun Y. (2024). The Concept of Levels of Detail for 3D Niche Models in CityGML. ISPRS Ann. Photogramm. Remote Sens. Spat. Inf. Sci..

[22] Biljecki F., Ledoux H., Stoter J. (2016). An improved LOD specification for 3D building models. Comput. Environ. Urban Syst..

[23] Biljecki F., Lim J., Crawford J., Moraru D., Tauscher H., Konde A., Adouane K., Lawrence S., Janssen P., Stouffs R. (2021). Extending CityGML for IFC-sourced 3D city models. Autom. Constr..

[24] Saeidian B., Rajabifard A., Atazadeh B., Kalantari M. (2023). A semantic 3D city model for underground land administration: Development and implementation of an ADE for CityGML 3.0. Tunn. Undergr. Space Technol..

[25] Biljecki F., Kumar K., Nagel C. (2018). CityGML Application Domain Extension *ADE*: Overview of developments. Open Geospat. Data Softw. Stand..

[26] Di Stefano F., Malinverni E.S. (2025). Extension of the CityGML Schema for a New Model of Accessible Urban Environment. Int. Arch. Photogramm. Remote Sens. Spat. Inf. Sci..

[27] Gürsoy Sürmeneli H., Koeva M., Alkan M. (2022). The Application Domain Extension (ADE) 4D Cadastral Data Model and Its Application in Turkey. Land.

[28] Chadzynski A., Krdzavac N., Farazi F., Lim M.Q., Li S., Grisiute A., Herthogs P., von Richthofen A., Cairns S., Kraft M. (2021). Semantic 3D City Database — An enabler for a dynamic geospatial knowledge graph. Energy AI.

[29] (2021). OGC City Geography Markup Language (CityGML) Part 1: Conceptual Model Standard.

[30] (2025). OGC IndoorGML 2.0 Part 1—Conceptual Model.

[31] Yao Z., Nagel C., Kunde F., Hudra G., Willkomm P., Donaubauer A., Adolphi T., Kolbe T.H. (2018). 3DCityDB—A 3D Geodatabase Solution for the Management, Analysis, and Visualization of Semantic 3D City Models Based on CityGML. Open Geospat. Data Softw. Stand..

[32] Zeeshan H.M.A., Faizan M., Zia U., Gohar A. RAG Powered LLMs for QA: Evolution, Challenges, Applications, and Future Directions. Proceedings of the 2025 International Conference on Communication Technologies (ComTech).

[33] Gao Y., Xiong Y., Gao X., Jia K., Pan J., Bi Y., Dai Y., Sun J., Wang M., Wang H. (2024). Retrieval-Augmented Generation for Large Language Models: A Survey. arXiv.

[34] Shahade A.K., Deshmukh P.V. Enhancing Natural Language Processing: A Comprehensive Review of Retrieval Augmented Generation. Proceedings of the 2024 4th International Conference on Sustainable Expert Systems (ICSES).

[35] Yu H.Q., McQuade F. (2025). RAG-KG-IL: A Multi-Agent Hybrid Framework for Reducing Hallucinations and Enhancing LLM Reasoning through RAG and Incremental Knowledge Graph Learning Integration. arXiv.

[36] Wahidur R.S.M., Kim S., Choi H., Bhatti D.S., Lee H.N. (2025). Legal Query RAG. IEEE Access.

[37] Khan F. (2025). Building a Self-Improving Agentic RAG System. https://levelup.gitconnected.com/building-a-self-improving-agentic-rag-system-f55003af44c4.

[38] Ko K., Nyein T.Y., Oo K.K., Oo T.Z., Zin T.T. Retrieval Augmented Generation for Document Query Automation using Open source LLMs. Proceedings of the 2024 5th International Conference on Advanced Information Technologies (ICAIT).

[39] Budakoglu G., Emekci H. (2025). Unveiling the Power of Large Language Models: A Comparative Study of Retrieval-Augmented Generation, Fine-Tuning, and Their Synergistic Fusion for Enhanced Performance. IEEE Access.

[40] Yang C.C.Y., Liu G.J., Yu C.J. (2025). Optimizing RAG in Programming Education: A Comparative Study of Models and Strategies. IEEE Access.

[41] Suryavanshi K., Thikekar N., Pawar R., Ashtekar S. Implementation of RAG Based Question-Answering Application. Proceedings of the 2025 International Conference on Data Science and Business Systems (ICDSBS).

[42] Song M. (2025). Enhancing RAG Performance by Representing Hierarchical Nodes in Headers for Tabular Data. IEEE Access.

[43] Flores N., Ramirez C., Mauricio D. (2025). Expert Multi-Agent Conversational System Using Retrieval-Augmented Generation and Dynamic Text-to-SQL for Government Transparency. IEEE Access.

[44] Jang H., Yu K., Park S. (2023). Managing 3D GIS Data for Indoor Environment Using Property Graph Database. IEEE Access.

[45] Ding L., Xiao G., Pano A., Fumagalli M., Chen D., Feng Y., Calvanese D., Fan H., Meng L. (2023). Integrating 3D City Data through Knowledge Graphs. arXiv.

[46] Kanna K., Kolbe T.H. (2025). Advanced User Interaction with Urban Digital Twins using Large Language Models. ISPRS Ann. Photogramm. Remote Sens. Spat. Inf. Sci..

[47] Zhao Y., Xu K., Zhu Z., Hu Y., Zheng Z., Chen Y., Ji Y., Gao C., Li Y., Huang J. (2025). CityEQA: A Hierarchical LLM Agent on Embodied Question Answering Benchmark in City Space. arXiv.

[48] Conesa J., Mula F., Contero M., Camba J. (2023). A multi-agent framework for collaborative geometric modeling in virtual environments. Eng. Appl. Artif. Intell..

[49] Giusti L., Werner O.A., Taiello R., Costa M.C., Tosun E., Protani A., Molina M., de Almeida R.L., Cacace P., Santos D.R. (2025). Federation of Agents: A Semantics-Aware Communication Fabric for Large-Scale Agentic AI. arXiv.

[50] Zhang S., Huang M., Liu S., Meng F., Xie Y., Ren X., Zhang Y., Shao W. (2025). AI-Driven Post-Earthquake Emergency Material Demand Prediction: Integrating RAG With Reasoning Large Language Model. IEEE Access.

[51] Azmi R., Abdellahi E.A.S., Bounabi M., Chenal J., Hlal M., Diop E. Towards a Multi-Agent System Based on LLM and RAG for Automated and Customizable Urban Diagnostics. Proceedings of the 2025 International Conference on Intelligent Systems: Theories and Applications (SITA).

[52] Li Z., Ning H. (2023). Autonomous GIS: The next-generation AI-powered GIS. Int. J. Digit. Earth.

[53] Akinboyewa T., Li Z., Ning H., Lessani M.N. (2025). GIS Copilot: Towards an autonomous GIS agent for spatial analysis. Int. J. Digit. Earth.

